# FindSim: A Framework for Integrating Neuronal Data and Signaling Models

**DOI:** 10.3389/fninf.2018.00038

**Published:** 2018-06-26

**Authors:** Nisha A. Viswan, Gubbi Vani HarshaRani, Melanie I. Stefan, Upinder S. Bhalla

**Affiliations:** ^1^National Centre for Biological Sciences, Bangalore, India; ^2^Tata Institute of Fundamental Research, The University of Trans-Disciplinary Health Sciences and Technology, Bangalore, India; ^3^Centre for Discovery Brain Sciences, University of Edinburgh, Edinburgh, United Kingdom; ^4^ZJU-UoE Institute, Zhejiang University, Hangzhou, China

**Keywords:** simulation, signaling pathway, systems biology, biochemistry, pharmacology, LTP, synaptic signaling

## Abstract

Current experiments touch only small but overlapping parts of very complex subcellular signaling networks in neurons. Even with modern optical reporters and pharmacological manipulations, a given experiment can only monitor and control a very small subset of the diverse, multiscale processes of neuronal signaling. We have developed FindSim (Framework for Integrating Neuronal Data and SIgnaling Models) to anchor models to structured experimental datasets. FindSim is a framework for integrating many individual electrophysiological and biochemical experiments with large, multiscale models so as to systematically refine and validate the model. We use a structured format for encoding the conditions of many standard physiological and pharmacological experiments, specifying which parts of the model are involved, and comparing experiment outcomes with model output. A database of such experiments is run against successive generations of composite cellular models to iteratively improve the model against each experiment, while retaining global model validity. We suggest that this toolchain provides a principled and scalable way to tackle model complexity and diversity of data sources.

## Introduction

Neuronal signaling is a complex, multiscale phenomenon which includes genetic, biochemical, transport, structural, protein synthesis, electrical and network components. There is an abundance of models of specific parts of this landscape, with a special focus on electrophysiological properties of neurons (Hodgkin and Huxley, 1952; Bhalla and Bower, 1993; De Schutter and Bower, 1994; Narayanan and Johnston, 2010) and biochemical signaling in plasticity (Lisman, 1985; Bhalla and Iyengar, 1999; Shouval et al., 2002; Hayer and Bhalla, 2005; Smolen et al., 2006; Kim et al., 2010; Manninen et al., 2010; Li et al., 2012; Stefan et al., 2012). Each of these models has its own parameterization idiosyncrasies, and even when the data sources are described in some detail (e.g., Bhalla and Iyengar, 1999) the derivation of specific rate terms and parameters is something of an individualistic art form. Further, each of these models typically incorporates far more knowledge about the biological system than is apparent from a plain listing of data sources. While this has resulted in high quality, hand-crafted models for specific processes, there are several major drawbacks of this almost universal modeling process. First, it is idiosyncratic. Second, most models are highly specific for individual questions posed by the developers. Third, by necessity, all such models are tiny subsets of known signaling (Heil et al., 2018). Fourth, models rarely venture across scales, that is cross electrical and biochemical, or structural and genetic.

There are some counter-currents to this highly personalized modeling process. The first has been the emergence of a range of standards for model specification (Hucka et al., 2003; Gleeson et al., 2010), experiments (Waltemath et al., 2011; Garcia et al., 2014; Teeters et al., 2015), and model output (Ray et al., 2016). The continued development of community-based standards is overseen by the COMBINE initiative (Hucka et al., 2015). These standards mean that even though individual model development may remain personalized, models can be much more readily shared. Numerous databases now host such models (Migliore et al., 2003; Sivakumaran et al., 2003; HarshaRani et al., 2005; Le Novère et al., 2006; Gleeson et al., 2015). With this set of resources, the models, simulation experiments performed on them, and their output, can each be specified in a platform-neutral and unambiguous manner.

A second recent development has been the emergence of simulators designed for multiscale signaling (Ray and Bhalla, 2008; Wils and De Schutter, 2009) as well as the incorporation of multiscale features in existing simulators (Bhalla, 2002b; Brown et al., 2011; McDougal et al., 2013). With these developments the most common scale crossover, between spatially detailed electrical and chemical models, is greatly facilitated.

A third major counter-current to this highly personalized modeling process is the development of model specification pipelines. The CellML project has developed data pipelines for model composition, annotation, model reduction and linkage to databases (Beard et al., 2009). The Allen Brain Project (Gouwens et al., 2018) and the Human Brain Project (Markram et al., 2015) have each developed systematic approach to parameterizing neuronal models, and the availability of such open resources has enabled development of independent efforts for experiment-driven modeling workflows (Stockton and Santamaria, 2017). These models build on previously developed ion-channel specifications and the parameter tuning is typically by way of assigning experimentally-driven passive properties and scaling channel densities, both in reduced and in detailed cellular morphologies. There are several related approaches to specify experimental data and metadata. For example, Silva and colleagues (Silva and Müller, 2015; Matiasz et al., 2017) have come up with frameworks for defining neurobiological experiments. Much more structured experiments such as microarrays (Brazma et al., 2001), next-generation sequencing, e.g., (Kent et al., 2010) or proteomics (Taylor et al., 2006, 2007) have their own metadata formats. In neuroscience, several such initiatives exist, for various types of neurobiological data (Garcia et al., 2014; Rübel et al., 2016; Stead and Halford, 2016). These specification formats are very powerful ways to ensure experimental consistency and reproducibility. However, our objectives were distinct, and more restricted, in two important ways. First, we needed not to reproduce experiments, but to be able to map them to simulations. Second, we needed to do this for a wide range of “legacy” style experiments, where structured metadata was neither available, nor easily specified. We therefore selected a small core subset of metadata and experimental data of direct relevance to simulation development.

The current study examines how to systematically use experimental data to parameterize multiscale neuronal signaling models reproducibly, scalably, openly, and in a generally applicable manner. It is clearly desirable to have a standard for facile mapping between experiments and models, especially in the rapidly expanding domain of neural physiology and signaling. We envision the role of FindSim as a first key step towards a standard, by demonstrating a functional implementation of experiment-driven simulation specification in a production environment. We examine the requirements for such an eventual standard by exploring a diverse and challenging set of use cases. We report two core developments: how to unambiguously and scalably match experimental observations to models, and how to manage development of very large models having thousands of components needing thousands of experimental constraints. Both are combined in FindSim, the Framework for Integration of Neuronal Data and SIgnaling Models. We explain FindSim and illustrate a model development pipeline capable of handling such models and their associated experiments.

## Methods and Results

### General Approach

**Figure 1:**
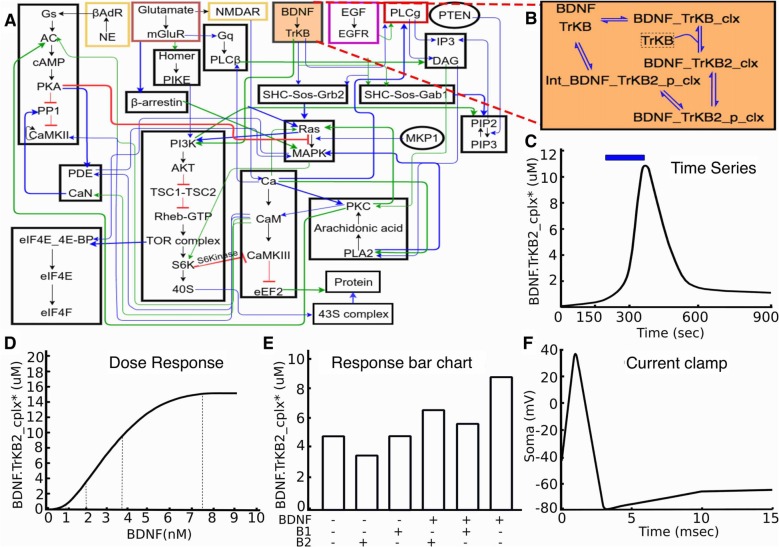
Signaling model and experiments on it. **(A)** Block diagram of model. **(B)** Expanded reaction scheme for one of the reaction blocks. The experiments typically act on similar small subsets of the full model. **(C–F)**: Typical kinds of experiments on the model. **(C)** Time-series experiment with stimulus pulse (brain-derived neurotrophic factor (BDNF), blue) leading to signaling response (TrkB receptor activation, black). **(D)** Dose-response experiment, where defined BDNF stimuli lead to receptor activation. **(E)** Schematic of drug interaction experiment, where different combinations of stimuli are examined for their response, as shown by the bar chart. **(F)** Schematic voltage trace following a step current clamp stimulus.

We illustrate our approach using a large core model of biochemical signaling which is designed to be embedded in a single-compartment electrical model (Figure 1). The biochemical model has over 300 molecular species and a similar number of reactions and is drawn from several neuronal signaling models (Bhalla and Iyengar, 1999; Hayer and Bhalla, 2005; Jain and Bhalla, 2009). While large by current standards, this model is, of course, far from the current known complexity of synaptic signaling (Bayés et al., 2011; Heil et al., 2018). Even though reduced, the current models explore many of the technical challenges for model specification that will arise in more complete future models and serve as a good test-bed for the current analysis.

Based on experience with development of neuronal signaling models, both within our groups and from the published literature, we chose three categories of experiments for our initial set. These were time-series, dose-response and multi-stimulus response. It was our observation that a large fraction (typically well over half) of data panels from the articles that were used for prior model development studies in this domain (Bhalla and Iyengar, 1999; Shouval et al., 2002; Lindskog et al., 2006; Stefan et al., 2008; Kim et al., 2010) fell into these three categories. As such, these were easy targets with substantial value for model development. Further, we were able to generalize effectively within each category. For example, there are many variations of dose-response experiments. These may use different initial conditions, different ways of controlling the stimulus (dose), absolute or relative scaling for measuring the response, and so on. These variations were readily accommodated within our framework. As we discuss below, this approach also generalizes to the electrophysiological domain, and commonly used current and voltage-clamp experiments also fall into this framework.

### Model Development and Parameterization Pipeline

The first of the core advances in this study is the definition of a model development and parameterization pipeline that takes the model and subjects it to a battery of experimental tests, defined in an open, extendable and structured form. In brief, any of a set of models is simulated according to instructions derived from the experimental dataset, and the outcome for each such simulation is scored according to how well the model fits the data. The models may be variants of the reference model, updated with progressively improved parameters or reaction schemes. They may also be specifically altered “mutant” or “disease” versions of the reference, for example, representing known mutants through the loss of a given molecular species. Our reference model is a composite of several modeling studies linked together based on known interactions.

**Figure 2:**
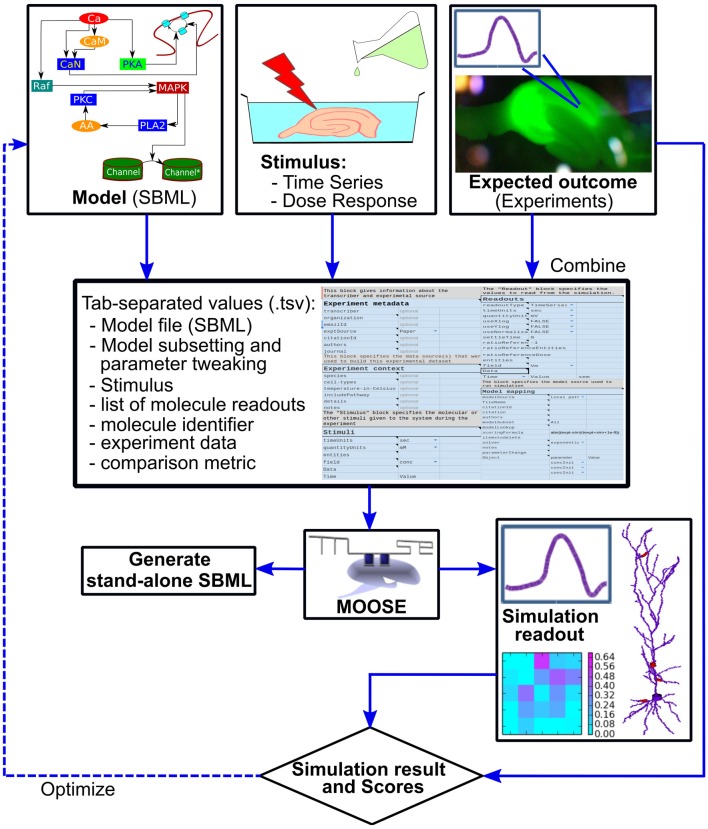
Block diagram of the FindSim pipeline. Top: the inputs to the pipeline. Left: Model specification, typically in SBML. Middle: experimental stimuli. Right: experimental outcomes. Middle from top: the two experimental inputs and the metadata for how to apply these to the model are specified in a structured experiment definition in a tab-separated text file (.tsv file). This may be manipulated by an enhanced spreadsheet, or through a GUI. Below: the experiment definition and model are read in and executed by a Python/MOOSE script. This may either run the simulation and compare with experiment (right, lowermost) or emit SBML output so that the experiment can be run on other simulators. There are options to utilize the score from the simulation comparison as part of a model optimization cycle.

**Figure 3:**
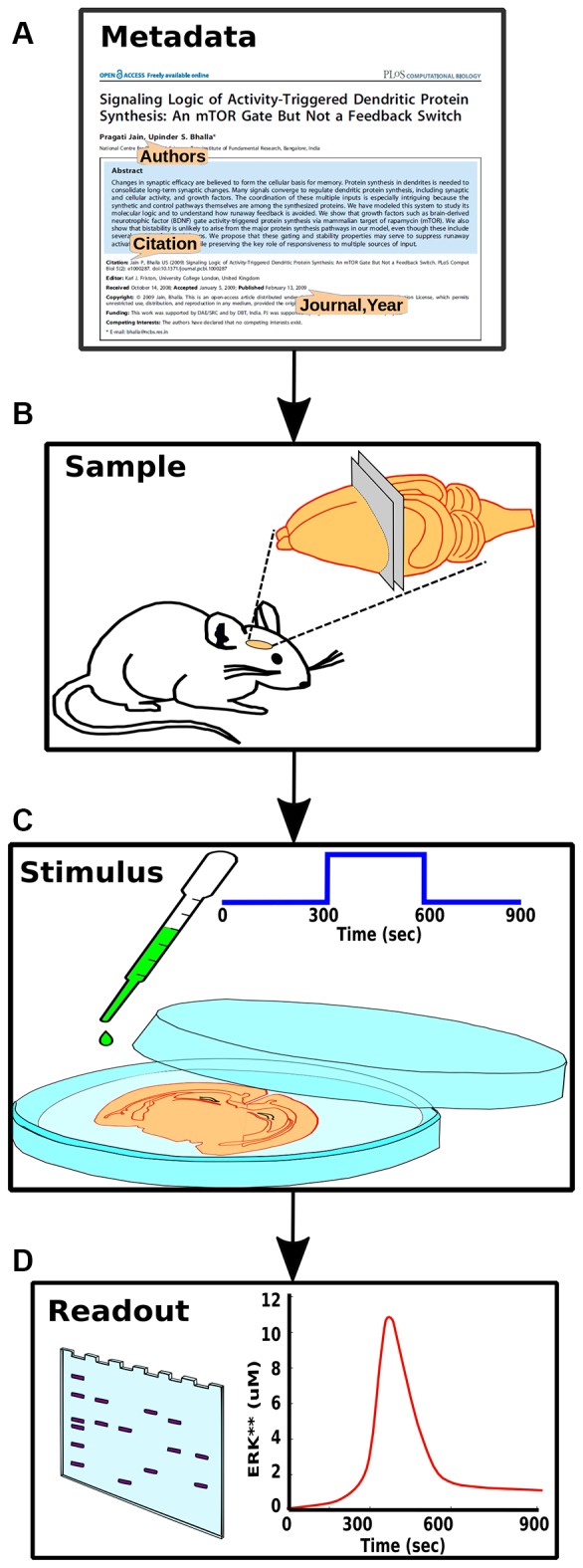
Components of experiment specification. **(A)** Experiment metadata, including citation information and authorship. **(B)** Experimental context, including species, preparation and conditions. **(C)** Stimulus information such as molecular identity and concentration of a pharmacological agent. **(D)** Readouts from the experiment, such as a gel or time-course, each representing a set of measurables that should have a direct mapping to the model.

Each structured experiment entry in the dataset is drawn from one of the three categories illustrated above: time-series, dose-response, or multi-stimulus response (Figure 1). The ~40 experiments in our initial database are all variants of these three categories, though further categories can readily be implemented. The experiment definitions specify which part of the model to use, which stimuli to deliver, and what results to expect (Figures 2, 3). The structured form of these definitions makes them independent of the exact model implementation.

To run through the models, we have implemented a Python-based script that reads the model and experiment definitions and launches the MOOSE simulator to execute the experiment. This wrapper script then examines the outputs from MOOSE and compares these with those expected from experiment (Figure 2). This comparison is scored according to a user-defined scoring function specified as part of the experiment definition. In order to improve cross-platform testability, the pipeline can also generate an SBML file for execution of the model on alternate platforms. This SBML file contains the model definition for that subpart or version of the reference model upon which the experiment is carried out, and where feasible, the definition of the stimulus that is applied to the model.

Stepping back, this entire pipeline can be run successively with different models, different experiments, and different scoring schemes. This is an embarrassingly parallel problem, so it is relatively easy to decompose the entire experiment set onto different processors on a cluster. Further, the structure of the scoring pipeline lends itself to an optimization step (dashed line in Figure 2) in which the model parameters are tweaked to improve the match to experiment as reflected in the model score.

In summary, we have implemented a model, a database and structure for experiment specification, and a pipeline to systematically test the model against each experiment. Each of these is in an open format and is accessible for other models and simulation tools^1^.

### Experiment Specification and Mapping

The second core development in this study is a methodology for mapping experiments to large models. The conceptual challenge is how to merge many pathway-specific readouts into a consistent, cell-wide model. One could do so either by assembly of small models into a large composite one, or by extraction of small sub-models from the reference composite model. The first approach maps closely to the individual experiments and modular perspectives of pathway function (Bhalla and Iyengar, 2001). We have described composition of large models from small modules for the first approach in previous work (Bhalla, 2002a), but with a topological rather than parameterization emphasis. The second approach incorporates interactions and takes a systems-level view.

The problems with the first approach are: (a) it is just as important and difficult to parameterize interactions *between* pathways as it is to parameterize the pathways themselves; (b) modifications to one pathway are likely to have knock-on effects on many others. The second approach (which we adopt here) handles pathway modularity by running the experiment on just that sub-portion of the model that is addressed in the experiment. It addresses point (a) by building in the pathway interactions into the composite model. This facilitates model comparison with experiments that span interacting pathways, and hence provides a process to parameterize the interactions. It does pose a specific technical issue of cleanly extracting small sub-models from the large one, which is addressed below. Point (b) remains relevant even in the second approach using a composite model. However, the larger model is amenable to “clean-up” of knock-on effects by running through all the subsequent experiments to fine-tune sub-models that may be impacted by the original change.

We now describe the structured experiment definition that implements the mapping of experiments to a large composite model (Figure 3). The goal of this definition is to provide a standardized, model-independent specification of experimental context, inputs, observed experimental results, and support for mapping each of these to model definitions. Some portions of such a definition have been formalized in the Simulation Experiment Definition Markup Language (Waltemath et al., 2011). Other aspects have been implemented in individual projects (Wolstencroft et al., 2017). SBML itself has support for delivery of specific inputs within the model definition markup file (Hucka et al., 2003). To our knowledge there is no unified specification standard that supports all of the elements essential to developing a model pipeline of the kind we envisage.

The key parts of the structured experiment definition are: (1) Experiment metadata. This specifies who did the experiment, citations, and other context. (2) Experiment context. This specifies species, cell-types, sample extraction methods, and the pathways expected to be relevant to the experiment. It also includes temperature, pH and other conditions pertinent to reproducibility. (3) Stimuli. These are the specific manipulations performed in the course of the experiment. This section can be quite diverse, and currently represents three main classes of experiments (Figure 1). For example, in the case of a time-series experiments, the inputs section would specify which molecule(s) were added to the preparation, at what times, and at what concentration. (4) Readouts. These are the readouts from the experimental preparation. This too is specific for each class of experiment. For example, in a time-series experiment the output would specify which molecule(s) were monitored, the observed concentration, and where available, the standard error for each observation.

**Figure 4:**
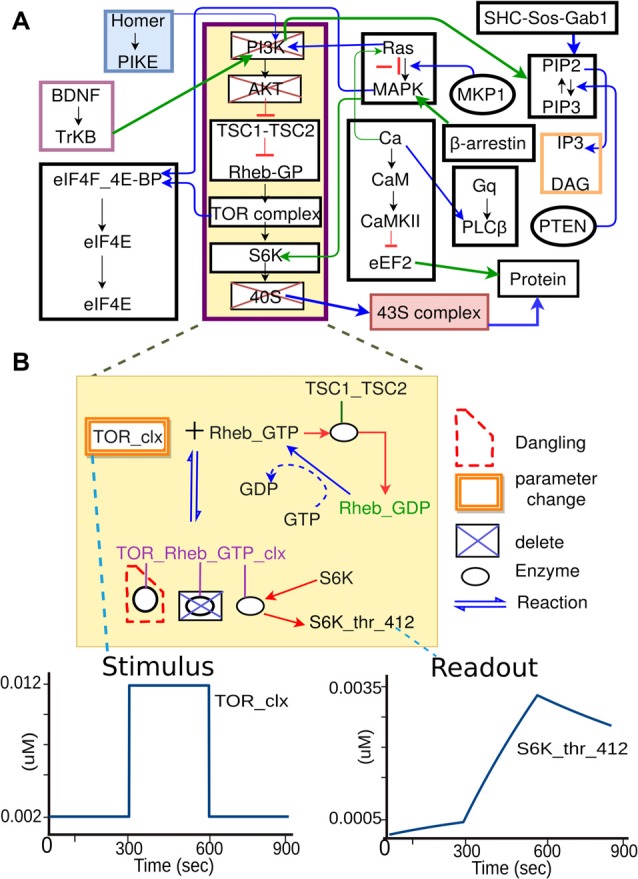
Specification of model sub-parts. **(A)** Original full model, from which a few pathways are selected. **(B)** Further selections of reactions from the subset of pathways. Deleted molecules, reactions, and enzymes are indicated by boxes with blue crosses. In some cases, deleting molecules leads to dangling reactions (red dashed boundary), which lack one or more reactants. The system identifies these. In addition to removal of extraneous reactions and reactants, the experiment specification may involve alteration of parameters, such as the concentration of a molecule that is buffered in the experiment. This is illustrated as orange boxes around the molecule TOR_clx. Finally, the model specification also defines the mapping between the experimental names for stimulus or readout molecules, to the corresponding names for these molecules as used in the model.

The above four sections are all purely in the experimental domain and could in principle be filled in without any reference to the modeling. Part 5 (Model mapping) is special in that it explicitly sets up the mapping of experimental entities to model entities. Several of these are straightforward, such as identifying the mapping between input/output molecules in the experiment and in the model. The crucial and novel part of the experiment definition is the extraction of the relevant pathways from the composite model. While the experimentalist will provide some indication of the relevant pathways when describing the experiment (Figure 3B), it requires some understanding of model structure to formally define which pathways should be used. In brief, we impose a hierarchical organization onto our reference composite models, thus facilitating grouping of molecules and reactions into pathways, and even further groupings of related pathways into larger pathway circuits. In most experiments, the extraction of sub-models is a simple matter of specifying which pathways need to be used. Further fine-tuning of the sub-model may involve addition or removal of specific molecules or reactions from the final subset for extraction (Figure 4). In the current implementation, the extracted subset is defined as a string in the familiar directory/file format, which is also similar to the XPATH format used in XML.

While this key step is conceptually simple, the implementation of pathway extraction has a number of subtleties (Figure 4B). First, there is the problem of “dangling” reactions, which occur when model extraction has removed one or more of the substrates of a reaction. This admits of a technical solution by way of explicit tests and warnings for such situations. Second, extracted pathways may lose key regulatory inputs, leading to uncontrolled build-up of signals at run-time. This requires human inspection of the outcome of each experiment, and subsequent reconsideration of the extraction procedure. Third, experimental conditions frequently modify the base model not just by removal of pathways, but also by addition of buffers and inhibitors to the medium. This is addressed by expansion of Part 5 of the experiment specification to include such manipulations. Fourth, the mapping of experiment to model entities is not always clean. For example, there may be multiple protein isoforms in the experiment, the model, or both. In essence, this is a problem of model detail, and the modeler and experimentalist have to get together to decide the appropriate mapping, given the detail in any given composite model.

One of the key design decisions for the current pipeline implementation was* not* to try to automate too much of the mapping of entities that comprises part 5 of the experiment definition. For example, one could envisage using extensive Gene Ontology (GO) markup of each pathway or molecule to automatically obtain the appropriate mapping between experiment and model (Ashburner et al., 2000). This is the approach taken in existing tools for model merging (Neal et al., 2015), model feature extraction (Alm et al., 2015; Neal et al., 2015), or combination of models with experimental datasets (Cooper et al., 2011) based on semantic annotation. All those tools rely on high-quality expert annotation. For our purposes, our analysis was that the GO, or any other markup, would typically fall short of specifying all the details of experiments or model implementation. Thus, in practice one would almost always have to layer on further explicit specification of entities, and thus have to fall back on some more complicated version of our current “part 5”. We also felt that extensive GO annotation from the outset would impose a further burden on the experimentalist as well as on anyone adapting existing models.

This effectively means that part 5 of the experiment definition requires curation by human experts. This is an opportunity for experimentalists and modelers to collaborate and provides a framework for clarifying assumptions on both sides and reaching agreement on the model. It is also worth noting that once this work has been done once for a specific combination of sub-model and experiment, it can be used for testing and validating future versions of the model.

**Figure 5:**
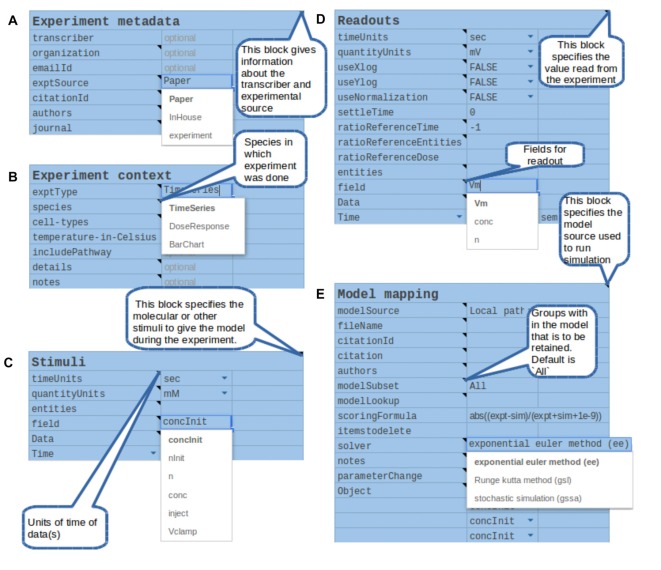
Components of the structured specification of experiment and its mapping to the simulation, implemented as a spreadsheet. In all sections the top line specifies a block of data, and the left column specifies fields to fill in that block. All fields and block titles support tool-tips, that is, pop-up help windows with an explanation of the block and field. These are illustrated here as speech balloons. Fields having a restricted set of options, such as quantity units, are specified with pull-down menus. In several cases there are tabulated sections, which contain value-range limited entries and which can be extended for additional data points. **(A)** Experiment metadata section. This specifies data about the experiment source and who transcribed it. A menu item is illustrated for the “exptSource” field. **(B)** Experiment context section. This specifies biological context for the experiment. **(C)** Stimuli. This section specifies inputs that were given during the experiment: which entity or molecule to change, which parameter was altered, and finally a series of time-value pairs that specifies the stimulus. **(D)** Readouts. This specifies which entities (such as molecules) were monitored during the experiment, and what values were obtained at each readout time. It may include error bars for each value. **(E)** Model mapping. This section is the only model-specific part. It indicates a reference model for which the experiment was first tested. For that model it specifies how to obtain the appropriate subset of pathways, molecules and other model entities to use in the simulated “experiment”. The model map next specifies which numerical methods to use. There follows a dictionary of entity names, which maps the experimenter’s naming scheme to unique entity names in the simulation. Finally, there is a table of parameters that have to be changed so that the model matches the experimental conditions. For example, some of the molecules in the experiment may now be buffered to specified values.

Having determined the components of the structured experiment specification, we next explain its implementation. Drawing upon lessons from existing projects that implement some parts of these requirements (Wolstencroft et al., 2011, 2017), we chose an enhanced spreadsheet interface as our initial interface (Figure 5). Our interface is implemented and exported in Google Docs^2^ and additional versions are provided for Microsoft Excel and Open Office. The contents of these spreadsheets are exported to tab-separated value (tsv) files for use by FindSim. We provide a schema for these tsv files^3^.

There were several reasons for a first implementation as a spreadsheet. First, spreadsheets are easy to set up and familiar to users. Our spreadsheet interface supports key features such as bounds checking on entered data, for example to ensure that only positive values are used. It also supports pull-down menu options for restricted choices, such as concentration units. Explanatory tool-tips are readily incorporated to provide immediate online help. Spreadsheets are inherently extendable with additional data rows or columns and can easily export data into the standard tab-separated value (tsv) format we use for driving the simulations. Finally, spreadsheets are highly portable, including in the cloud.

Based on a pilot set of ~40 experiments, we have found that a large range of biochemical experiments can be specified with just three kinds of very similar spreadsheets: time-series, dose-response, and multi-stimulus response. In all cases there are identical panels for experiment metadata and context. There are slightly specialized blocks for experimental stimulus and readouts. The final, model mapping panel (Figure 5E) is again almost identical. This framework is easily extended to further kinds of experiments, including electrophysiological and imaging data.

In summary, we have designed and implemented a framework for specifying the design and outcomes of experiments in a form which maps directly to corresponding simulation experiments. This supports validation, scoring and optimization of models. The key innovations are formalization of a wide range of experiments and a procedure for defining how to extract parts of a large model that are necessary and sufficient to account for any given experiment.

### Example of Data Flow Through the Pipeline

**Figure 6:**
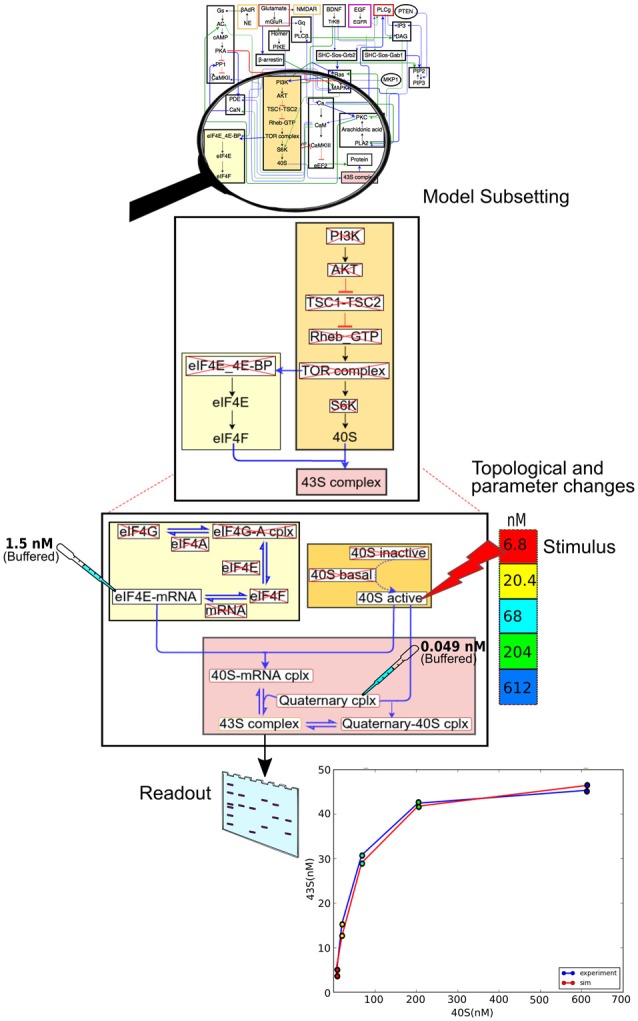
Data flow using the model specification, experiment specification, model subset extraction, simulation and comparison with output.

We now illustrate the data flow through the pipeline (Figure 2) using a specific example of a pre-existing model, and an experiment to be applied to it (Figure 6). The stages in the pipeline are:

1. Model specification. In this case the model specification is a pre-existing SBML file.
2. Experimental details. The experiment is a straightforward stimulus-response experiment in which the 40S subunit of the translation complex is applied to a solution with a known amount of eIF4E-mRNA, and the formation of 43S subunit is monitored.
3. Mapping between experiment and model. As the experimental pathways are a small subset of the larger model, we select a few relevant pathways, and further we remove from the pathway models those reactions that are not present in the experiment.
4. Simulation control. Here we take those molecules that are buffered in the experiment and change the model accordingly. We then run the simulation, applying the stimulus to one of the molecules at the specified times.
5. We now compare experiment and simulation readouts, using a scoring equation defined in the model mapping section from Figure 5.
6. At this stage we could use the score to do a local optimization of parameters using manual or automated optimization. This would give us a version of the main model where the local parameters for this pathway have been matched to experiment.
7. We now repeat steps 2 to 6 for different experiments, to obtain a global score for the model. Additionally, local optimizations will need validation from experiments that involve larger subsets of the overall model.

At the end of this process, we will have a model that is a better fit to specific experiments, and we also have a score that can be given to the model as a whole. Note that it is entirely up to the modeler-experimentalist team to decide how much weight should be given to different experiments.

### Cross-Experiment Model Reproducibility

A key goal of the FindSim pipeline is to expose models to a range of experiments so that the model is a good fit to all of them, not just a single case. This is a particularly tough constraint when we have multiple experiments that probe responses of the same and overlapping signaling pathways. In this section we describe how the model database can include just this kind of overlapping experiment, to show how the modeler and experimentalist can together examine reproducibility and generalizability of the core model.

**Figure 7:**
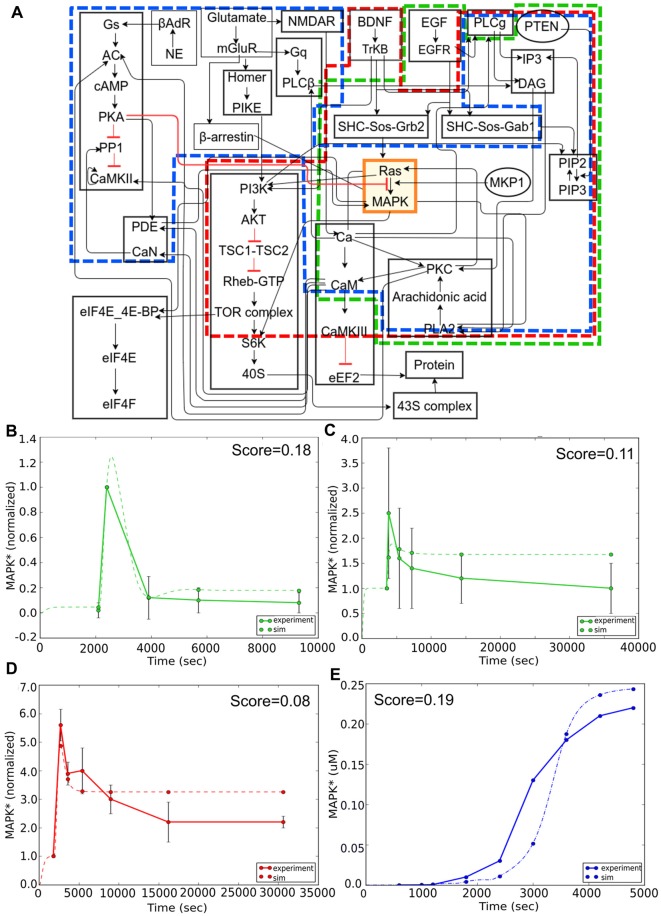
Reproducibility example: multiple experimental inputs converging onto a common signaling pathway (MAPK). **(A)** Block diagram of composite model. Sub-models for the different inputs are indicated in green (epidermal growth factor, EGF), red (BDNF) and blue (Calcium). **(B)** Response to EGF stimulus is a transient activation of MAPK. Model (dashed green line) closely follows experimental curve (solid green line). **(C)** Response to EGF in similar preparation but lower dose. Note that the simulated peak response is higher than experiment in **(B)**, but lower in **(C)**, leading to difficulties in model fitting. **(D)** Response to BDNF stimulus is also a transient, but the time-course of MAPK signaling in this experiment is much longer than it was in panel **(B)**. **(E)** Response to an LTP-induction stimulus for calcium, consisting of three pulses separated by 600 s. Here the reference is an earlier simulation predicting sustained activation of MAPK. Remarkably, there is a reasonable match to the reference behavior in all three cases, despite the inputs converging onto the MAPK pathway through different signaling pathways, and the results drawn from very different data sources.

Here we focused our attention on the MAPK signaling pathway. We first considered reproducibility of the core part of this pathway, in which MAPK is stimulated by an epidermal growth factor (EGF) signal in PC12 cells (Figure 7A, green boundary; Teng et al., 1995). The simulation approximates the experiment quite closely (Figure 7B). We next illustrate a fundamental limitation on being able to reproducibly fit data: the observation that different experiments with very similar contexts may give mutually inconsistent results (Figures 7B,C). We then considered experiments involving overlap of the core (MAPK) pathway but distinct input signaling via brain-derived neurotrophic factor (BDNF) in E18 primary embryonic hippocampal neurons (Ji et al., 2010). In the case of BDNF, the core model behavior of a transient strong response followed by a sustained low response was preserved, but the time-courses and the intervening stages were quite different. Again, the model performed reasonably well in comparison to this experiment (Figure 7D). This was reassuring because it meant that the same core pathway generalized well for two completely different kinds of input. In these two experiments the stimulus and model system are the same, but there is a difference in dose. Even allowing for differences in effective dose, the simulation cannot fit both of these. As discussed above, the FindSim framework provides for a user-defined scoring scheme for each experiment, so that broader considerations can factor into how the user weights each experiment.

Finally, for Ca^2+^ input we asked if the model could replicate the qualitative behavior of sustained activity, that had been predicted in an earlier modeling study (Bhalla and Iyengar, 1999). Again, this activated a large number of distinct input stages but the core MAPK pathway was able to replicate the previous behavior of switching to a state of sustained high activity following three calcium pulses which corresponded to three tetanic stimuli used for LTP induction (Figure 7E). Overall, this exercise showed the efficacy of the FindSim framework in testing model reproducibility across quite different stimulus conditions.

## Discussion

We have developed FindSim, a framework for systematic, data-driven construction of large biologically detailed models of neuronal signaling. The key advances are: (1) A simulation pipeline that combines a database of structured experimental data with each model, to systematically generate scores of how well the model fits the entire dataset. (2) A way to systematically specify and extract small sub-parts of the full model upon which to carry out these simulated experiments. Together with the underlying Python-driven MOOSE simulation engine for multiscale models, this framework is an open, standards-driven, and scalable approach to developing reliable, large-scale models.

### Big Models and Biological Problems

It is widely accepted that complex biological pathways benefit from structured modeling approaches to address the many routes by which signals flow between stimuli and physiological outcomes (Kitano, 2002; Hunter and Borg, 2003). This is particularly relevant for complex neurogenetic diseases such as autism, where mutations in key signaling components cause ramifying perturbations in many pathways. The complexity of this problem is exacerbated by cellular homeostasis mechanisms, which may lead to partial rescue of some symptoms, but not others. The expectation is that as models begin to incorporate the relevant range of pathways, these outcomes may be better understood. Further, such models would be excellent platforms upon which to conduct tests of possible pharmacological and other manipulations with the goal of suggesting treatments (Rajasethupathy et al., 2005).

### Correctness of Big Models

A common criticism of big models, dating from the von Neumann tradition, is that they have so many parameters that the modeler could do anything (such as fit an elephant) with them. Here we first explain how the current model building pipeline counters this criticism. We then point to two ways in which the details embedded in a big model improve its testability and utility.

The modeling framework described in this study provides a systematic way to avoid the problems of multi-parameter models. Here we have formalized how multiple experiments map to different parts of the model. Further, this formalization facilitates parameterization, and testing, from individual reactions, to multi-pathway cascades. By testing the models at many scales of function, this approach is able to ensure not only that individual pathways work as observed, but that they work together in a manner consistent with experiment. This process has been adopted, albeit in a more free-form manner, for other large models (Bhalla and Iyengar, 1999; Karr et al., 2012).

There are two key positive aspects that large, detailed models bring to ascertaining correctness. The first is that there is a clear, usually one-to-one mapping between experimental entities (molecules, reactions) and their model counterparts. Thus, there is no ambiguity about what each readout represents. The second major positive of detail is that the curse of the abstract model—that it may abstract away essential functional detail—is avoided. A further, empirically noted corollary of having biologically detailed models is that they tend to partake of similar kinds of functional robustness as their biological counterparts (Morohashi et al., 2002). In biology this means that minor fluctuations in metabolism or protein distribution has little functional effect. In the model this brings the additional benefit that it tends to behave well even if the parameters are, inevitably, somewhat off. Thus, the methodology of the current study is designed to allow principled construction of large, detailed models that avoid the major drawbacks of such models, while benefiting from their advantages.

### Big Data and Big Models

Our appreciation for biological complexity has risen steeply with the flood of large-scale data. As an example in neuronal signaling, we now know the identities of some 1500 postsynaptic proteins (Bayés et al., 2011), but our understanding of synaptic function has not kept pace with this explosion of data. Models have long been tools for understanding complex systems, as well as predicting their properties in health and disease (Kitano, 2002; Rajasethupathy et al., 2005). A systematic alignment of big data to developing big models is therefore highly desirable. One of the major problems with doing this is that a large fraction of current experiments is better at providing model *constraints* rather than model *parameters*. A model constraint is an observation that the model must satisfy, but it does not always yield easily usable data for improving the model. For improving models, one needs experiments that more directly provide parameters.

Automated parameter estimation and tuning is important for developing large, complex models. The current framework is designed to efficiently run many simulated experiments on a model, and this is of obvious utility for parameter estimation and tuning. This is a complex and well-studied topic (Chou and Voit, 2009; Geier et al., 2012; Sun et al., 2012) and is out of scope of the current report. A key feature of the FindSim framework is that every run computes a score of how well the model output fits the experiment. Effective scoring is itself closely linked to details of parameter optimization. Here we simply give the user freedom to specify arbitrary mathematical expressions for comparison of model output to experimental output, including error bars (example scoring formula in Figure 5E). This expression can also include less-tangible scaling factors based on the judgment of the team implementing the test, such as the reliability of the experimental approach, or whether the experimental system was a mouse or a rat.

Model constraints typically arise from remote input-output relationships. A peculiarity of biological signaling systems is that very long chains of elementary events, such as reactions, may link stimulus and response. For example, long-term potentiation (LTP), which is the staple of synaptic plasticity studies, is mechanistically separated from synaptic input patterns by at least the following steps: presynaptic calcium events, neurotransmitter release, synaptic channel opening, calcium influx, activation of kinase pathways, protein synthesis and receptor translocation (Bliss and Collingridge, 1993). Each of these steps may involve numerous biophysical events and chemical reactions. Yet at an observational level, LTP is reliable, easily measured, and well characterized. It is an excellent model constraint. From a modeling viewpoint, there are far more “good” experiments on LTP, than there are measurements of mechanisms of just one of its steps: dendritic protein synthesis. Big data is therefore of limited value for big models unless the experiments are designed to home in on, and parameterize, finer mechanistic steps. In the context of our modeling pipeline, experiments on small pathways are better for parameterization, including the use of optimization. Long-pathway experiments tell us what the overall model should do, but don’t directly help us refine it. Thus, our data-model development framework defines the kinds of big data that are of most relevance to constructing reliable, big models.

### Scalability

The current report describes the core concepts and implementation of a first level modelling and data organization effort for models of neuronal signaling. The approach is designed to be scalable both in the kinds of problems it can take on, and in the technical capabilities it brings to the table.

The current framework was designed around studies of autism spectrum disorders, with the technical aim of building sufficiently detailed models so as to be able to match up with the wide range of current data. Thus only a few models were initially envisioned: a control model, and a few with known disease-causing mutations. The approach is readily extended to many other neurodevelopmental and other diseases provided there are clear molecular signatures of the signaling deficit in each case.

An obvious further extension of the approach is to apply it to different cell-types, and in parallel to develop experiment libraries to parameterize them. In addition to neurons, it would be interesting to model glia, and then proceed to making models not just of individual neurons but small groupings of strongly coupled cells in neural tissue.

Scalability can also be envisaged as extending the experiment-model interplay to different physical processes. From the viewpoint of neuronal function, it is clearly important to also consider the domain of electrical activity of neurons. A few simulators (e.g., NEURON, MOOSE, STEPS) are now able to simultaneously model electrical and chemical signaling, but each has different ways to specify such multiscale models (Ray and Bhalla, 2008; Wils and De Schutter, 2009; McDougal et al., 2013). There are efforts to broaden model standards to include chemical as well as electrical signaling (Cannon et al., 2014). While the evolution of the FindSim framework to such models is beyond the scope of the current article, as proof of principle we illustrate the use of the FindSim format on the Hodgkin-Huxley model of an action potential^4^. This requires very minor extensions within the framework of a time-series experiment. We anticipate that an important direction for the FindSim framework will be to support multiscale experiments that synthesize electrophysiological stimuli with multiple signaling and physiological readouts.

A further aspect of scalability is the ability of this framework to host competing models, in the sense that models are hypotheses of neuronal signaling function. Here the value of the open experimental database becomes evident. Different groups can readily re-assign weights and scoring terms for different experiments, to develop models that better fit their interpretation of the experimental literature. The expectation is that such competing models would spur the execution of more definitive experiments to decide between the alternatives, and thus advance the field.

On the technical side, there are clear directions with respect to the evolution of the experiment specification format, including standards development for storing them in databases.

The current experiment specification is set up through a spreadsheet and stored in tab-separated value (tsv) format. Clearly a more flexible and powerful format would be desirable as we scale up to much larger models and datasets. We have considered extensions to the extant SED-ML standard (Waltemath et al., 2011) as one possible way to define the experiments. Another alternative may be JSON and its associated schema (Crockford, 2006). Each of these is also much better suited to being handled in a database. On the interface front it would be desirable to develop a browser-based graphical interface to the model/experiment building pipeline, where the runs may be hosted in the cloud. These all lend themselves to incorporation into the FindSim framework. In summary, the FindSim framework is a principled, scalable framework that lends itself to reproducibly integrating experiments with complex multiscale models of neuronal signaling systems.

The FindSim framework currently relies on human interactions between modelers and experimentalists for the “model mapping” (part 5 of the pipeline). This was a conscious choice on our part. This stage of the framework is an ideal place for encouraging interaction between human experts, since this is a stage that relies on expert judgment on what the various parts of a model and of an experiment mean. In terms of scalability, this may be a bottleneck. Indeed, other initiatives have aimed at automating similar processes (Cooper et al., 2011; Alm et al., 2015; Neal et al., 2015). It should be noted, however, that those automations depend on high-quality annotations. As such, they do not eliminate the need for human curation, it just happens at a different stage of the process (model/data annotation). Which of those two approaches is ultimately better scalable, and to what extent the expert annotation component can be automated, remains an interesting avenue for future research.

## Datasets

The datasets and code used for this study can be found in https://github.com/BhallaLab/FindSim. The MOOSE simulator is hosted at https://moose.ncbs.res.in/ and on https://github.com/BhallaLab/moose.

## Author Contributions

NV built the model, assembled the database of experimental conditions, designed the experiment interface and worked on the figures. GVHR worked on the code, designed the experiment interface and on the figures. MS examined existing model development projects, helped conceptualize the framework and wrote the article. UB worked on the code, designed the project and experiment interface and wrote the article.

## Conflict of Interest Statement

The authors declare that the research was conducted in the absence of any commercial or financial relationships that could be construed as a potential conflict of interest.

## Footnotes

^1^https://github.com/BhallaLab/FindSim

^2^https://www.ncbs.res.in/faculty/bhalla-findsim/worksheet

^3^https://github.com/BhallaLab/FindSim

^4^https://github.com/BhallaLab/FindSim

## References

[B2] Alm R. Waltemath D. Wolfien M. Wolkenhauer O. Henkel R. (2015). Annotation-based feature extraction from sets of SBML models. J. Biomed. Semantics 6:20. 10.1186/s13326-015-0014-4 25904997 PMC4405863

[B3] Ashburner M. Ball C. A. Blake J. A. Botstein D. Butler H. Cherry J. M.. (2000). Gene ontology: tool for the unification of biology. The gene ontology consortium. Nat. Genet. 25, 25–29. 10.1038/75556 10802651 PMC3037419

[B4] Bayés A. van de Lagemaat L. N. Collins M. O. Croning M. D. R. Whittle I. R. Choudhary J. S.. (2011). Characterization of the proteome, diseases and evolution of the human postsynaptic density. Nat. Neurosci. 14, 19–21. 10.1038/nn.2719 21170055 PMC3040565

[B5] Beard D. A. Britten R. Cooling M. T. Garny A. Halstead M. D. B. Hunter P. J.. (2009). CellML metadata standards, associated tools and repositories. Philos. Trans. A Math. Phys. Eng. Sci. 367, 1845–1867. 10.1098/rsta.2008.0310 19380315 PMC3268215

[B6] Bhalla U. S. (2002a). The chemical organization of signaling interactions. Bioinformatics 18, 855–863. 10.1093/bioinformatics/18.6.855 12075021

[B7] Bhalla U. S. (2002b). Use of kinetikit and GENESIS for modeling signaling pathways. Methods Enzymol. 345, 3–23. 10.1016/s0076-6879(02)45003-3 11665614

[B8] Bhalla U. S. Bower J. M. (1993). Exploring parameter space in detailed single neuron models: simulations of the mitral and granule cells of the olfactory bulb. J. Neurophysiol. 69, 1948–1965. 10.1152/jn.1993.69.6.1948 7688798

[B9] Bhalla U. S. Iyengar R. (1999). Emergent properties of networks of biological signaling pathways. Science 283, 381–387. 10.1126/science.283.5400.381 9888852

[B10] Bhalla U. S. Iyengar R. (2001). Functional modules in biological signalling networks. Novartis Found. Symp. 239, 4–13; discussion 13–15, 45–51. 10.1002/0470846674.ch2 11529315

[B11] Bliss T. V. Collingridge G. L. (1993). A synaptic model of memory: long-term potentiation in the hippocampus. Nature 361, 31–39. 10.1038/361031a0 8421494

[B12] Brazma A. Hingamp P. Quackenbush J. Sherlock G. Spellman P. Stoeckert C.. (2001). Minimum information about a microarray experiment (MIAME)-toward standards for microarray data. Nat. Genet. 29, 365–371. 10.1038/ng1201-365 11726920

[B13] Brown S.-A. Moraru I. I. Schaff J. C. Loew L. M. (2011). Virtual NEURON: a strategy for merged biochemical and electrophysiological modeling. J. Comput. Neurosci. 31, 385–400. 10.1007/s10827-011-0317-0 21340454 PMC3221887

[B14] Cannon R. C. Gleeson P. Crook S. Ganapathy G. Marin B. Piasini E.. (2014). LEMS: a language for expressing complex biological models in concise and hierarchical form and its use in underpinning NeuroML 2. Front. Neuroinform. 8:79. 10.3389/fninf.2014.00079 25309419 PMC4174883

[B15] Chou I.-C. Voit E. O. (2009). Recent developments in parameter estimation and structure identification of biochemical and genomic systems. Math. Biosci. 219, 57–83. 10.1016/j.mbs.2009.03.002 19327372 PMC2693292

[B16] Cooper J. Mirams G. R. Niederer S. A. (2011). High-throughput functional curation of cellular electrophysiology models. Prog. Biophys. Mol. Biol. 107, 11–20. 10.1016/j.pbiomolbio.2011.06.003 21704062

[B17] Crockford D. (2006). The application/json Media Type for JavaScript Object Notation (JSON). Available online at: https://tools.ietf.org/html/rfc4627 [Accessed May 15, 2018].

[B18] De Schutter E. Bower J. M. (1994). An active membrane model of the cerebellar Purkinje cell. I. Simulation of current clamps in slice. J. Neurophysiol. 71, 375–400. 10.1152/jn.1994.71.1.375 7512629

[B19] Garcia S. Guarino D. Jaillet F. Jennings T. R. Pröpper R. Rautenberg P. L.. (2014). Neo: an object model for handling electrophysiology data in multiple formats. Front. Neuroinformatics 8:10. 10.3389/fninf.2014.00010 24600386 PMC3930095

[B20] Geier F. Fengos G. Felizzi F. Iber D. (2012). Analyzing and constraining signaling networks: parameter estimation for the user. Methods Mol. Biol. 880, 23–39. 10.1007/978-1-61779-833-7_2 23361979

[B21] Gleeson P. Crook S. Cannon R. C. Hines M. L. Billings G. O. Farinella M.. (2010). NeuroML: a language for describing data driven models of neurons and networks with a high degree of biological detail. PLoS Comput. Biol. 6:e1000815. 10.1371/journal.pcbi.1000815 20585541 PMC2887454

[B22] Gleeson P. Silver A. Cantarelli M. (2015). “Open source brain,” in Encyclopedia of Computational Neuroscience (New York, NY: Springer.), 2153–2156.

[B23] Gouwens N. W. Berg J. Feng D. Sorensen S. A. Zeng H. Hawrylycz M. J.. (2018). Systematic generation of biophysically detailed models for diverse cortical neuron types. Nat. Commun. 9:710. 10.1038/s41467-017-02718-3 29459718 PMC5818534

[B24] HarshaRani G. V. Vayttaden S. J. Bhalla U. S. (2005). Electronic data sources for kinetic models of cell signaling. J. Biochem. (Tokyo) 137, 653–657. 10.1093/jb/mvi083 16002985

[B25] Hayer A. Bhalla U. S. (2005). Molecular switches at the synapse emerge from receptor and kinase traffic. PLoS Comput. Biol. 1, 137–154. 10.1371/journal.pcbi.0010020 16110334 PMC1185646

[B26] Heil K. F. Wysocka E. Sorokina O. Kotaleski J. H. Simpson T. I. Armstrong J. D. (2018). Analysis of proteins in computational models of synaptic plasticity. BioRxiv [Preprint]. 10.1101/254094

[B27] Hodgkin A. L. Huxley A. F. (1952). A quantitative description of membrane current and its application to conduction and excitation in nerve. J. Physiol. 117, 500–544. 12991237 10.1113/jphysiol.1952.sp004764 PMC1392413

[B28] Hucka M. Finney A. Sauro H. M. Bolouri H. Doyle J. C. Kitano H.. (2003). The systems biology markup language (SBML): a medium for representation and exchange of biochemical network models. Bioinformatics 19, 524–531. 10.1093/bioinformatics/btg015 12611808

[B29] Hucka M. Nickerson D. P. Bader G. D. Bergmann F. T. Cooper J. Demir E.. (2015). Promoting coordinated development of community-based information standards for modeling in biology: the COMBINE initiative. Front. Bioeng. Biotechnol. 3:19. 10.3389/fbioe.2015.00019 25759811 PMC4338824

[B30] Hunter P. J. Borg T. K. (2003). Integration from proteins to organs: the physiome project. Nat. Rev. Mol. Cell Biol. 4, 237–243. 10.1038/nrm1054 12612642

[B31] Jain P. Bhalla U. S. (2009). Signaling logic of activity-triggered dendritic protein synthesis: an mTOR gate but not a feedback switch. PLoS Comput. Biol. 5:e1000287 10.1371/journal.pcbi.1000287 19242559 PMC2647780

[B32] Ji Y. Lu Y. Yang F. Shen W. Tang T. T.-T. Feng L.. (2010). Acute and gradual increases in BDNF concentration elicit distinct signaling and functions in neurons. Nat. Neurosci. 13, 302–309. 10.3410/f.3559973.3266072 20173744 PMC4780419

[B33] Karr J. R. Sanghvi J. C. Macklin D. N. Gutschow M. V. Jacobs J. M. Bolival B.. (2012). A Whole-cell computational model predicts phenotype from genotype. Cell 150, 389–401. 10.1016/j.cell.2012.05.044 22817898 PMC3413483

[B34] Kent W. J. Zweig A. S. Barber G. Hinrichs A. S. Karolchik D. (2010). BigWig and BigBed: enabling browsing of large distributed datasets. Bioinforma. Oxf. Engl. 26, 2204–2207. 10.1093/bioinformatics/btq351 20639541 PMC2922891

[B35] Kim M. Huang T. Abel T. Blackwell K. T. (2010). Temporal sensitivity of protein kinase a activation in late-phase long term potentiation. PLoS Comput. Biol. 6:e1000691. 10.1371/journal.pcbi.1000691 20195498 PMC2829045

[B36] Kitano H. (2002). Systems biology: a brief overview. Science 295, 1662–1664. 10.1126/science.1069492 11872829

[B37] Le Novère N. Bornstein B. Broicher A. Courtot M. Donizelli M. Dharuri H.. (2006). BioModels Database: a free, centralized database of curated, published, quantitative kinetic models of biochemical and cellular systems. Nucleic Acids Res. 34, D689–D691. 10.1093/nar/gkj092 16381960 PMC1347454

[B38] Li L. Stefan M. I. Novère N. L. (2012). Calcium input frequency, duration and Aamplitude differentially modulate the relative activation of calcineurin and CaMKII. PLoS One 7:e43810. 10.1371/journal.pone.0043810 22962589 PMC3433481

[B39] Lindskog M. Kim M. Wikström M. A. Blackwell K. T. Kotaleski J. H. (2006). Transient calcium and dopamine increase PKA activity and DARPP-32 phosphorylation. PLoS Comput. Biol. 2:e119. 10.1371/journal.pcbi.0020119 16965177 PMC1562452

[B40] Lisman J. E. (1985). A mechanism for memory storage insensitive to molecular turnover: a bistable autophosphorylating kinase. Proc. Natl. Acad. Sci. U S A 82, 3055–3057. 10.1073/pnas.82.9.3055 2986148 PMC397705

[B41] Manninen T. Hituri K. Kotaleski J. H. Blackwell K. T. Linne M.-L. (2010). Postsynaptic signal transduction models for long-term potentiation and depression. Front. Comput. Neurosci. 4:152. 10.3389/fncom.2010.00152 21188161 PMC3006457

[B42] Markram H. Muller E. Ramaswamy S. Reimann M. W. Abdellah M. Sanchez C. A.. (2015). Reconstruction and simulation of neocortical microcircuitry. Cell 163, 456–492. 10.1016/j.cell.2015.09.029 26451489

[B43] Matiasz N. J. Wood J. Wang W. Silva A. J. Hsu W. (2017). Computer-aided experiment planning toward causal discovery in neuroscience. Front. Neuroinform. 11:12. 10.3389/fninf.2017.00012 28243197 PMC5304468

[B44] McDougal R. A. Hines M. L. Lytton W. W. (2013). Reaction-diffusion in the NEURON simulator. Front. Neuroinformatics 7:28. 10.3389/fninf.2013.00028 24298253 PMC3828620

[B45] Migliore M. Morse T. M. Davison A. P. Marenco L. Shepherd G. M. Hines M. L. (2003). ModelDB: making models publicly accessible to support computational neuroscience. Neuroinformatics 1, 135–139. 10.1385/ni:1:1:135 15055399 PMC3728921

[B46] Morohashi M. Winn A. E. Borisuk M. T. Bolouri H. Doyle J. Kitano H. (2002). Robustness as a measure of plausibility in models of biochemical networks. J. Theor. Biol. 216, 19–30. 10.1006/jtbi.2002.2537 12076125

[B47] Narayanan R. Johnston D. (2010). The h current is a candidate mechanism for regulating the sliding modification threshold in a BCM-like synaptic learning rule. J. Neurophysiol. 104, 1020–1033. 10.1152/jn.01129.2009 20554832 PMC2934916

[B48] Neal M. L. Carlson B. E. Thompson C. T. James R. C. Kim K. G. Tran K.. (2015). Semantics-based composition of integrated cardiomyocyte models motivated by real-world use cases. PLoS One 10:e0145621. 10.1371/journal.pone.0145621 26716837 PMC4696653

[B49] Rajasethupathy P. Vayttaden S. J. Bhalla U. S. (2005). Systems modeling: a pathway to drug discovery. Curr. Opin. Chem. Biol. 9, 400–406. 10.1016/j.cbpa.2005.06.008 16006180

[B50] Ray S. Bhalla U. S. (2008). PyMOOSE: Interoperable Scripting in Python for MOOSE. Front. Neuroinform. 2:6. 10.3389/neuro.11.006.2008 19129924 PMC2614320

[B51] Ray S. Chintaluri C. Bhalla U. S. Wójcik D. K. (2016). NSDF: neuroscience simulation data format. Neuroinformatics 14, 147–167. 10.1007/s12021-015-9282-5 26585711 PMC4823348

[B52] Rübel O. Dougherty M. Prabhat Denes P. Conant D. Chang E. F.. (2016). Methods for specifying scientific data standards and modeling relationships with applications to neuroscience. Front. Neuroinform. 10:48. 10.3389/fninf.2016.00048 27867355 PMC5095137

[B53] Shouval H. Z. Bear M. F. Cooper L. N. (2002). A unified model of NMDA receptor-dependent bidirectional synaptic plasticity. Proc. Natl. Acad. Sci. U. S. A. 99, 10831–10836. 10.1073/pnas.152343099 12136127 PMC125058

[B54] Silva A. J. Müller K.-R. (2015). The need for novel informatics tools for integrating and planning research in molecular and cellular cognition. Learn. Mem. 22, 494–498. 10.1101/lm.029355.112 26286658 PMC4561409

[B55] Sivakumaran S. Hariharaputran S. Mishra J. Bhalla U. S. (2003). The Database of Quantitative Cellular Signaling: management and analysis of chemical kinetic models of signaling networks. Bioinformatics 19, 408–415. 10.1093/bioinformatics/btf860 12584128

[B56] Smolen P. Baxter D. A. Byrne J. H. (2006). A model of the roles of essential kinases in the induction and expression of late long-term potentiation. Biophys. J. 90, 2760–2775. 10.1529/biophysj.105.072470 16415049 PMC1414565

[B57] Stead M. Halford J. J. (2016). Proposal for a standard format for neurophysiology data recording and exchange. J. Clin. Neurophysiol. 33, 403–413. 10.1097/WNP.0000000000000257 26808620 PMC4956586

[B58] Stefan M. I. Edelstein S. J. Le Novère N. (2008). An allosteric model of calmodulin explains differential activation of PP2B and CaMKII. Proc. Natl. Acad. Sci. U S A 105, 10768–10773. 10.1073/pnas.0810309105 18669651 PMC2504824

[B59] Stefan M. I. Marshall D. P. Novère N. L. (2012). Structural analysis and stochastic modelling suggest a mechanism for calmodulin trapping by CaMKII. PLoS One 7:e29406. 10.1371/journal.pone.0029406 22279535 PMC3261145

[B60] Stockton D. B. Santamaria F. (2017). Integrating the allen brain institute cell types database into automated neuroscience workflow. Neuroinformatics 15, 333–342. 10.1007/s12021-017-9337-x 28770487 PMC5671885

[B61] Sun J. Garibaldi J. M. Hodgman C. (2012). Parameter estimation using meta-heuristics in systems biology: a comprehensive review. IEEE/ACM Trans. Comput. Biol. Bioinform. 9, 185–202. 10.1109/tcbb.2011.63 21464505

[B62] Taylor C. F. Hermjakob H. Julian R. K. Garavelli J. S. Aebersold R. Apweiler R. (2006). The work of the human proteome organisation’s proteomics standards initiative (HUPO PSI). OMICS 10, 145–151. 10.1089/omi.2006.10.145 16901219

[B63] Taylor C. F. Paton N. W. Lilley K. S. Binz P.-A. Julian R. K. Jones A. R.. (2007). The minimum information about a proteomics experiment (MIAPE). Nat. Biotechnol. 25, 887–893. 10.1038/nbt1329 17687369

[B64] Teeters J. L. Godfrey K. Young R. Dang C. Friedsam C. Wark B.. (2015). Neurodata without borders: creating a common data format for neurophysiology. Neuron 88, 629–634. 10.1016/j.neuron.2015.10.025 26590340

[B65] Teng K. K. Lander H. Fajardo J. E. Hanafusa H. Hempstead B. L. Birge R. B. (1995). v-Crk modulation of growth factor-induced PC12 cell differentiation involves the Src homology 2 domain of v-Crk and sustained activation of the Ras/mitogen-activated protein kinase pathway. J. Biol. Chem. 270, 20677–20685. 10.1074/jbc.270.35.20677 7657647

[B66] Waltemath D. Adams R. Bergmann F. T. Hucka M. Kolpakov F. Miller A. K.. (2011). Reproducible computational biology experiments with SED-ML–the simulation experiment description markup language. BMC Syst. Biol. 5:198. 10.1186/1752-0509-5-198 22172142 PMC3292844

[B67] Wils S. De Schutter E. (2009). STEPS: modeling and simulating complex reaction-diffusion systems with python. Front. Neuroinform. 3:15. 10.3389/neuro.11.015.2009 19623245 PMC2706651

[B68] Wolstencroft K. Krebs O. Snoep J. L. Stanford N. J. Bacall F. Golebiewski M.. (2017). FAIRDOMHub: a repository and collaboration environment for sharing systems biology research. Nucleic Acids Res. 45, D404–D407. 10.1093/nar/gkw1032 27899646 PMC5210530

[B69] Wolstencroft K. Owen S. Horridge M. Krebs O. Mueller W. Snoep J. L.. (2011). RightField: embedding ontology annotation in spreadsheets. Bioinformatics 27, 2021–2022. 10.1093/bioinformatics/btr312 21622664

